# Supplementary material for: Test@Work Texts: Mobile Phone Messaging to Increase Awareness of HIV and HIV Testing in UK Construction Employees during the COVID-19 Pandemic
Source: Int J Environ Res Public Health. 2020 Oct 26;17(21):7819. doi: 10.3390/ijerph17217819 (PMC7672579; doi:10.3390/ijerph17217819)
Supplement: Supplementary file 1 [file ijerph-17-07819-s001.zip › Supplementary Table S1_Final message set mapped to COM-B constructs.pdf]

Supplementary Table S1. Final message set mapped to COM-B constructs

Week	Text No		Message	COM-B construct*
	1	1	Welcome to Test@Work thank you for taking part! You'll receive 2-3 texts per week for the next 10 weeks with useful links and information. At the end we will send 4 questions you can text a reply to (standard rate). Messages will start this week and you can opt out at any time. If you receive all the texts and reply to all 4 messages you'll be entered into a prize draw to win £100 Amazon vouchers Optout: (text number)	
1	2	H1	High blood pressure puts you at risk of heart attacks and strokes. Get it checked regularly at pharmacies and your GP surgery. More info https://tx.vc/r/1hbs7<br><br>https://www.bhf.org.uk/informationsupport/risk-factors	
	3	S2	HIV is a virus that attacks the body.You can have it but show no symptoms.Without treatment you will develop AIDS.Get tested and treated https://tx.vc/r/1hb7c<br><br>https://www.nhs.uk/conditions/hiv-and-aids/	CPs, MR
2	4	H2	Stress, money, family and work can all affect our mental health, don't forget the page in your pack or take the MOOD QUIZ here https://tx.vc/r/1h5Bb<br><br>https://www.nhs.uk/conditions/stress-anxiety-depression/mood-self-assessment/	
	5	S3	Condoms are the best way to protect you and your partner from HIV and STI's they're FREE and come in different sizes, find where https://tx.vc/r/1hb8q<br><br>https://www.nhs.uk/service-search/other-services/Free%20condoms/LocationSearch/732	OS, CPs, OP, MR,
	6	H3	Do you have a healthy heart? How old is your heart? Find out your HEART AGE with this test https://tx.vc/r/1h5HX<br><br>https://www.nhs.uk/conditions/nhs-health-check/check-your-heart-age-tool/	
3	7	S4	HIV affects everyone, straight and gay. Not everyone with HIV gets symptoms but with early treatment you can live a normal healthy life. Get tested sh24.org.uk	CP, OS, MR
	8	H4	Weight loss contributes to lowering blood pressure and diabetes risk. There's lots of help to get started, with easy to follow diet plans https://tx.vc/r/1h5By<br><br>https://www.nhs.uk/live-well/healthy-weight/start-the-nhs-weight-loss-plan/	

	9	S5	20% of people in the UK who have HIV don't know it, they're at greatest risk of passing it on, get tested at least once a year to protect you and your partner.	MR, OS, CPs
4	10	H5	Want to know how you're doing? Take a look at your lifestyle including smoking drinking and diet with the How Are You Quiz https://tx.vc/r/1h5ED<br><br>https://www.nhs.uk/oneyou/how-are-you-quiz/	
	11	S6	HIV testing is confidential, easy and quick. No time? Or don't want to visit a clinic? you can order a FREE home testing kit here https://tx.vc/r/1hbdV	OS, OP, CP
5	12	H6	Anyone can be at risk of type 2 diabetes, the biggest factors are weight, fat around the belly and diet, find out how to prevent diabetes https://tx.vc/r/1h5Fb<br><br>https://www.diabetes.org.uk/preventing-type-2-diabetes	
	13	S7	Your HIV quick test can only check for infections in the last 90 days. Get tested every 3 months if you or your partner has unprotected sex.	MR, CPs, MA
6	14	H7	Looking to give up smoking or cut down? It's hard to do it alone, get your personal quit plan and download the smoke free app https://tx.vc/r/1h5Gg<br><br>https://www.nhs.uk/smokefree	
	15	S8	You can't tell someone has HIV or any other STI just by looking at them, use a condom and get tested regularly. For symptoms go to https://tx.vc/r/1hbeX<br><br>https://www.nhs.uk/conditions/hiv-and-aids/symptoms/	CPs, OS, MR
	16	H8	Everyone is affected by mental health, especially now. Stressed, anxious, just curious? Take the mind plan quiz for personalised advice https://tx.vc/r/1h5Hv<br><br>https://www.nhs.uk/oneyou/every-mind-matters/your-mind-plan-quiz/	
7	17	S9	Had unprotected sex? Sex whilst drunk or high? Worried you've been exposed to HIV? There are emergency treatment options visit https://tx.vc/r/1hbfB for more.<br><br>https://www.nhs.uk/conditions/hiv-and-aids/treatment/	MA, MR, OS
	18	H9	Want to cut down on the drink? Drink free days is a FREE app to help you track your drinking and can support you if you want to cut down https://tx.vc/r/1h5Ic<br><br>https://www.nhs.uk/oneyou/apps/	
8	19	S10	People who are being treated for HIV live normal life spans, have healthy sexual relationships and can have children without passing it on https://tx.vc/r/1hbfS	CPs, OS, MR, MA

			https://www.nhs.uk/conditions/hiv-and-aids/living-with/	
	20	H10	Want to try moving more? Try couch to 5K http://tiny.cc/Couch25K Not ready to run? Beginner guided workouts and apps that track your goals https://tx.vc/r/1hbjP<br><br>https://www.nhs.uk/oneyou/for-your-body/move-more/	
9	21	S11	Need advice on HIV or STI's? Where to get tested? How to protect yourself? There's lots of help from your local sexual health services http://tx.vc/r/1hbgd<br><br>https://www.nhs.uk/using-the-nhs/nhs-services/sexual-health-services/guide-to-sexual-health-services/	OP, CPs
	22	H11	Back pain is a very common problem in the UK. Here's some easy to follow exercises to help beat the pain and advice on back care https://tx.vc/r/1h5Jl<br><br>https://www.nhs.uk/live-well/exercise/lower-back-pain-exercises/	
10	23	S12	Did you know, you don't need to see your GP or be registered to get an HIV test? They also don't need to know if you test positive it can stay private.	OP, MR
	24	S13	Drug use is hard to talk about, injecting drugs puts you at risk of HIV, get tested often and find more or get help with addiction https://tx.vc.r/1hbh2<br><br>https://www.nhs.uk/live-well/healthy-body/drug-addiction-getting-help/	MA, OS
	25		Thanks for taking part in Test@Work, please help us by answering some final questions. You'll now receive 4 messages, reply to each question like a normal text.	
	26		1/4 Have you had an HIV test since your Test@Work day? Reply Yes or NO to this text.	
	27		2/4 Have you found these texts useful? Reply Yes or No	
	28		3/4 Have you learned anything new from these texts? Reply Yes or NO	
	29		4/4 Will you make any changes or take actions to look after your health as a result of these messages? Reply to tell us what.	

Note: *Behavioural mapping occurs for HIV awareness and testing messages

CPs=Capability Psychological, CP= Capability Physical OP= Opportunity Physical, OS =Opportunity Social, MR = Motivation Reflective, MA=Motivation Automatic
