# Supplementary material for: Test@Work Texts: Mobile Phone Messaging to Increase Awareness of HIV and HIV Testing in UK Construction Employees during the COVID-19 Pandemic
Source: Int J Environ Res Public Health. 2020 Oct 26;17(21):7819. doi: 10.3390/ijerph17217819 (PMC7672579; doi:10.3390/ijerph17217819)
Supplement: Supplementary file 1 [file ijerph-17-07819-s001.zip › Supplementary Table S2_Web links to HIV-related and health-related information.pdf]

Supplementary Table S2: Web links to HIV-related and health-related information

Message Number	HIV or Lifestyle	Web Link	Total number of clicks	% of delivered messages that generated impressions
2	L	https://www.bhf.org.uk/informationsupport/risk-factors	53	20.2
3	H	https://www.nhs.uk/conditions/hiv-and-aids/	39	15.2
4	L	https://www.nhs.uk/conditions/stress-anxiety-depression/mood-self-assessment/	37	14.6
5	H	https://www.nhs.uk/service-search/other-services/Free%20condoms/LocationSearch/732	45	17.9
6	L	https://www.nhs.uk/conditions/nhs-health-check/check-your-heart-age-tool/	44	17.7
8	L	https://www.nhs.uk/live-well/healthy-weight/start-the-nhs-weight-loss-plan/	31	12.6
10	L	https://www.nhs.uk/oneyou/how-are-you-quiz/	39	15.9
11	H	http://sh24.org.uk	59	24.1
12	L	https://www.diabetes.org.uk/preventing-type-2-diabetes	43	17.6
14	L	https://www.nhs.uk/smokefree	25	10.2
15	H	https://www.nhs.uk/conditions/hiv-and-aids/symptoms/	41	16.9
16	L	https://www.nhs.uk/oneyou/every-mind-matters/your-mind-plan-quiz/	29	11.9
17	H	https://www.nhs.uk/conditions/hiv-and-aids/treatment/	26	10.7
18	L	https://www.nhs.uk/oneyou/apps/	39	16.3
19	H	https://www.nhs.uk/conditions/hiv-and-aids/living-with/	21	8.8
20	L	https://www.nhs.uk/oneyou/for-your-body/move-more/	23	9.7
21	H	https://www.nhs.uk/using-the-nhs/nhs-services/sexual-health-services/guide-to-sexual-health-services/	24	10.8
22	L	https://www.nhs.uk/live-well/exercise/lower-back-pain-exercises/	29	12.4
24	H	https://www.nhs.uk/live-well/healthy-body/drug-addiction-getting-help/	22	9.4

Note: HIV: HIV prevention or testing-related; Lifestyle: general health and health behaviours. Links targeting a UK audience, valid at time of publication, last accessed [date 22/5/2020]
