# Supplementary material for: Test@Work Texts: Mobile Phone Messaging to Increase Awareness of HIV and HIV Testing in UK Construction Employees during the COVID-19 Pandemic
Source: Int J Environ Res Public Health. 2020 Oct 26;17(21):7819. doi: 10.3390/ijerph17217819 (PMC7672579; doi:10.3390/ijerph17217819)
Supplement: Supplementary file 1 [file ijerph-17-07819-s001.zip › Supplementary Table S4_Mobile health (mHealth) evidence reporting and assessment (mERA) Checklist.pdf]

Supplementary Table S4: Mobile health (mHealth) evidence reporting and assessment (mERA) Checklist.

**Criteria**	**Item no**	**Description**	**Page no. Where item is reported**
**1.1 Essential Criteria for all studies**			
INTRODUCTION			
**Rationale/scientific background**	1	Mentions previous studies or scientific documentation in a similar or different context, which have attempted to answer the question. States the rationale for the study.	3
**Objectives/ hypotheses**	2	Specific objectives of the study are stated. If a hypothesis is being tested- it is clearly stated.	4-5
**Logic Model/ Theoretical framework**	3	Theoretical framework/logic model depicts how the intervention influences the primary study outcome, and the pathway to the outcome. If the intervention is targeting behavior change, appropriate behavior change theory is described.	7-8
METHODS			
**Study design**	4	A description of the study design and how it was arrived at is presented	5-7
**Outcomes**	5	Clearly defined primary outcome measures to meet study objectives. Secondary outcome measures should be mentioned, if relevant.	10
**Data collection methods**	6	Description of data collection methods is provided. This could include description of the study tools/survey questionnaires/interview guides	9-10
**Participant eligibility**	7	Eligibility criteria for participants is described.	5
**Participant recruitment**	8	Description of how the study participants were recruited into the study. Might include self-selection, health facility based recruitment, community-based recruitment, among others.	5

**Bias**	9	The risk of biases is reported. Key examples of bias include recall bias (error in the accuracy or completeness of recollections), selection bias (error individuals or groups to take part), etc.	-
**Sampling**	10	How was the sample size determined? Was attrition accounted for, if relevant to study design?	5
**Setting and locations**	11	Description of the study population, as well as details on geographic area and context. Data could be collected in a sub-sample of a geographic area/ population where the intervention is being implemented. This concept refers to details on the population/context from which data were collected.	5
**Comparator**	12	Describes use of a comparison group. For studies that do not use a comparator, this would not be relevant. Please note that before/ after comparisons are also valid and should be noted.	-
**Data sources/measurement**	13	Describes the source of data for each variable of interest. Data source may include individuals, databases, search engines etc.	9-10
**Results**			
**Enrollment**	14	Enrollment procedures are described. The numbers of participants screened for eligibility, found to be eligible or not eligible, declined to be enrolled, and enrolled in the study are mentioned, as relevant	11
**Description of study population**	15	Demographic and/or clinical characteristics of participants in each study cohort are described	11-12
**Reporting on outcomes**	16	For each primary and secondary outcome, study findings are presented	12-14
**Discussion**			
**Summary of evidence**	17	General interpretation of the results in the context of current evidence and current theory is provided.	14-18
**Limitations**	18	Discussion of study limitations. This should address sources of potential biases and imprecision.	18

**Generalisability/external validity**	19	The applicability of study findings to other settings is discussed. Examples might include a discussion on study population, characteristics of the intervention, incentives, and compliance rates in other contexts.	16
**Conclusion/interpretation**	20	Interpretation of the results. Discussion of barriers and/or opportunities relating to policy, programs or research is included	20
**Conflicts**			
**Funding**	21	Sources of funding and role of funders is mentioned	21
**Ethical considerations**	22	The process of reviewing the ethical issues related to participant enrollment, obtaining consent and preservation of confidentiality are addressed	5
**Competing interests**	23	Clear description of conflict of interest, if any is mentioned	21
**1.2. Essential Criteria Based on Study Design**			
**1.2.1 Quantitative Criteria**			
**Confounding**	1	The risk of confounding and any methods used to address it are reported	-
**Statistical methods**	2	Methods for primary and additional analyses, such as subgroup analyses and adjusted analyses are described	10
**Missing data**	3	Methods for dealing with missing data due to incomplete surveys are reported. This refers to how the data for missing, non-response and other variables were handled.	-
**1.2.2 Qualitative Criteria**			
**Analytical methods**	1	Analytical methods are clearly described. In-depth description of the analysis process and how categories/themes were derived.	10
**Data validation**	2	Discusses use of triangulation, member checking (respondent validation), search for negative cases, or other procedures Reporting on cross verification from 2 or more sources, inter-rater reliability should be included, if relevant	-

**Reflexivity of account provided**	3	Researchers role and relationship to the respondent, wording and phrasing of questions and other factors that might have elicited a biased response are described	8
